# Shock Index as a Marker for Mortality Rates in Those Admitted to the Medical Intensive Care Unit from the Emergency Department

**DOI:** 10.7759/cureus.7903

**Published:** 2020-04-30

**Authors:** Nitasa Sahu, Stephanie Yee, Mukund Das, Shauna Trinh, Robert Amoruso, Mark Connolly, Anil Rama, Jamshed Zuberi

**Affiliations:** 1 Internal Medicine, Penn State Hershey Medical Center, Hershey, USA; 2 Surgery, St. Joseph's University Medical Center, Paterson, USA; 3 Internal Medicine, St. Joseph's University Medical Center, Paterson, USA; 4 Surgery, Riverside University Health System, Riverside, USA; 5 Sleep Medicine, Kaiser Permanente San Jose Medical Center, San Jose, USA

**Keywords:** shock, critical care, emergency department, mortality, medical intensive care unit (micu)

## Abstract

Objective

Shock index (SI) is defined as the heart rate divided by systolic blood pressure. Studies have shown a correlation between the shock index and mortality in trauma patients in prehospital settings and in the emergency department (ED). The objective of this study was to identify the utility of SI in predicting mortality in the medical intensive care unit (MICU) patients admitted from the ED and transfers from the floor to MICU.

Design

We performed a retrospective analysis of adult patients admitted to the MICU at our urban trauma hospital between January 2015 through August 2015 using ED vital signs to calculate the shock index and identify inpatient deaths. Similar data were examined for inpatient transfers to the MICU.

Results

Nine hundred and fifty patients were included in the study; 743 had an SI ≤ 0.99 with a mortality rate of 15.9%. Two hundred and seven patients had a SI ≥ 1.00 with a mortality rate of 22.7%. A higher SI was significant for mortality. There was no statistical significance in SI and mortality rate for patients transferred from the medical floor to the ICU.

Conclusions

Patients with an SI ≥ 1.00 from initial ED vital signs correlated with a higher mortality rate. In patients transferred from the floor to MICU, SI ≥ 1.00 did not correlate with a higher mortality rate.

## Introduction

The shock index (SI), defined as the heart rate divided by the systolic blood pressure, was first developed by Allgöwer and Buri in 1967 as a quick, noninvasive method to determine the degree of hypovolemia in hemorrhagic shock where the normal range is between 0.5 - 0.7 in healthy adults [1]. It was determined to be a more sensitive marker than each vital sign alone. Another study showed that a SI > 1.0 was associated with poorer outcomes in those with circulatory failure [2]. The study by Berger et al. validated a correlation for mortality in trauma patients where a SI > 0.9 identified the most critically ill patients despite normal vital signs [3]. The utility of SI in predicting mortality in the trauma population is also demonstrated in other studies where elevated SI > 1.3 is associated with increased mortality in patients with isolated torso injuries and prehospital SI > 0.9 predicts mortality in patients with polytrauma [4-6]. A review of the National Trauma Data Bank supported data that the shock index was a useful predictor of morbidity and mortality in geriatric trauma patients [7]. While several studies were done in trauma patients and the principles could theoretically be applied to those in other types of shock, only a few other studies have been done to assess all patients presenting to the Emergency Department (ED) to identify high-risk septic patients using the SI and lactate levels [8-9]. A study by Kaukonen et al. demonstrated that SI ≥ 1.0 correlated with the most specific predictor for mortality and high lactate levels [8]. Another study showed that patients with SI ≥ 0.7 were more likely to have sepsis hyperlactatemia, intensive care unit (ICU) admission, and mortality [9]. Both of these studies included only patients presenting to the ED. Our study evaluates the SI of all medical patients admitted through the ED going to the medical intensive care unit (MICU) and also identifies the mortality rate adjusted for beta-blocker use, which was a limiting factor in Berger’s study [3]. To further identify if the SI is a tool that can be used in deteriorating patients on the medical floor, all rapid response patients transferred to the MICU were taken into account as a separate analysis. 

## Materials and methods

This study is a retrospective analysis of patients admitted to the MICU at an urban hospital ranging in age from 20 - 101 years old using ED vital signs and identifying in-hospital deaths. Inclusion criteria were patients admitted to the MICU regardless of diagnosis. As expected, most patients admitted were older in age with a mean age of 63.3 years. Information on home medications (such as beta-blockers), albuterol, or recreational drugs (such as cocaine) was collected to see if those that could affect heart rates in either direction changed the results. A separate analysis was done to include all rapid responses from the medical floor that needed to be transferred to the ICU with an SI ≥ 1.00. No patients were excluded from analysis unless various data points of interest in the records were missing. 

## Results

**Figure 1:**
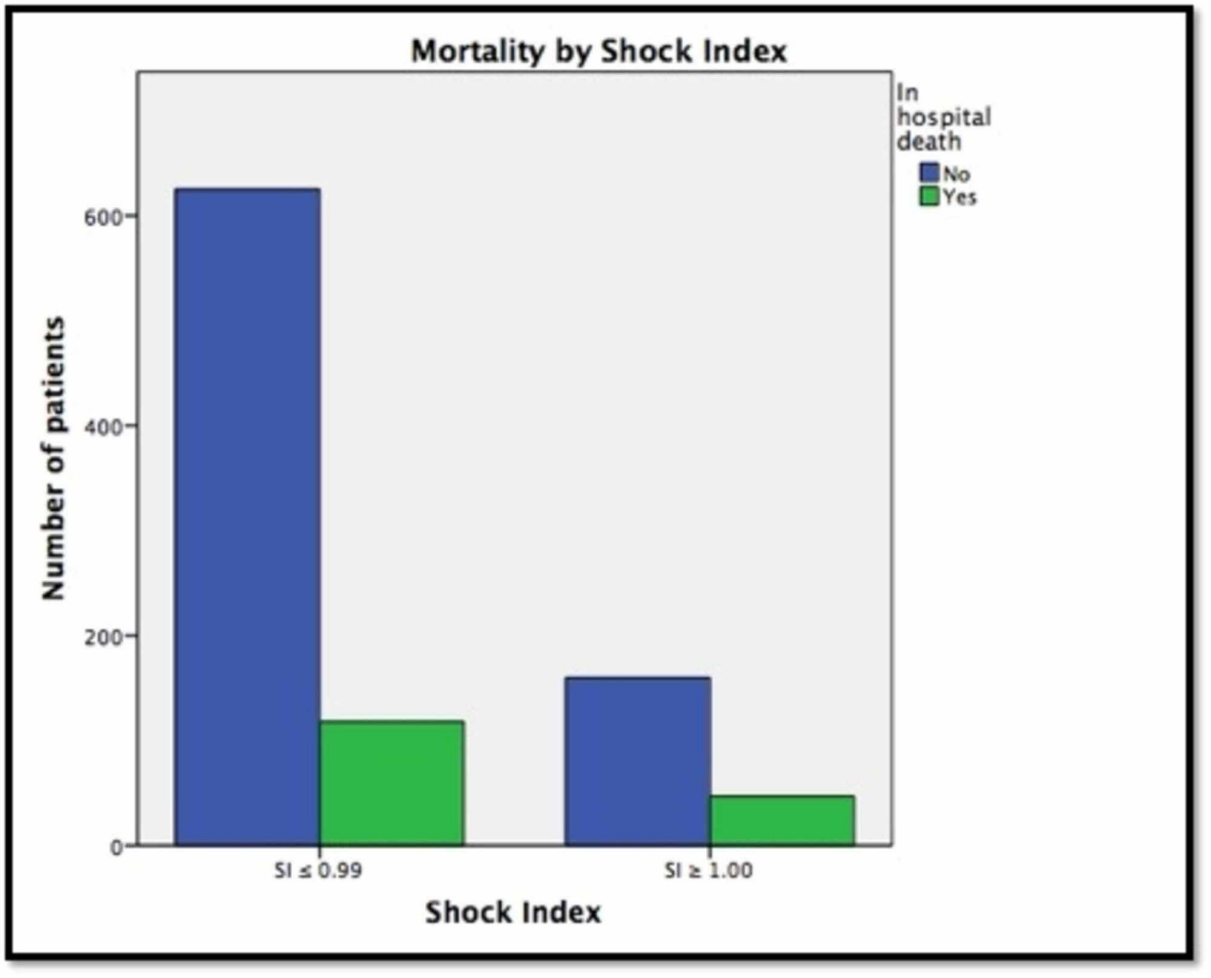
In-hospital deaths compared to admission shock index (SI)

**Figure 2:**
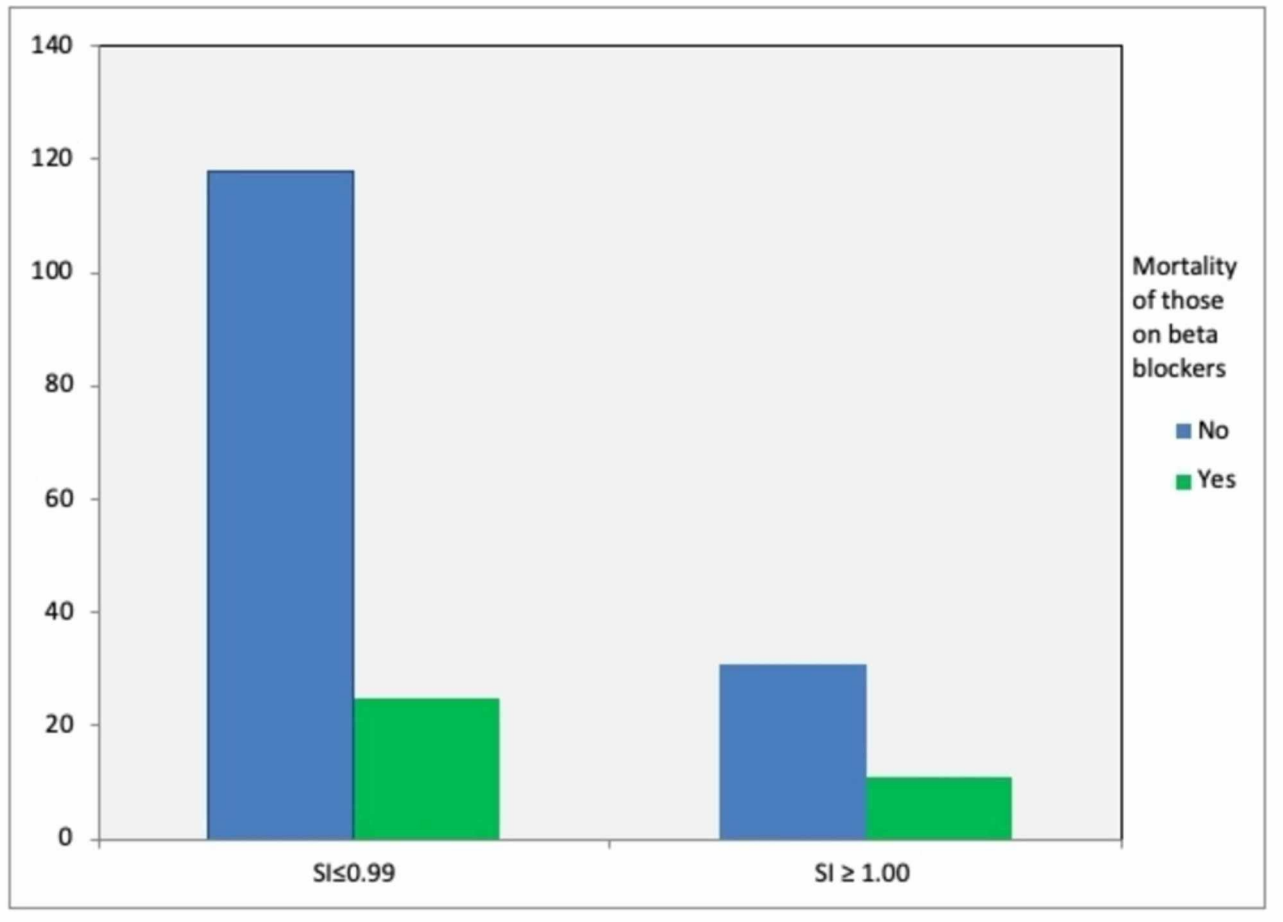
Shock index (SI) and mortality of those on beta-blockers

**Figure 3:**
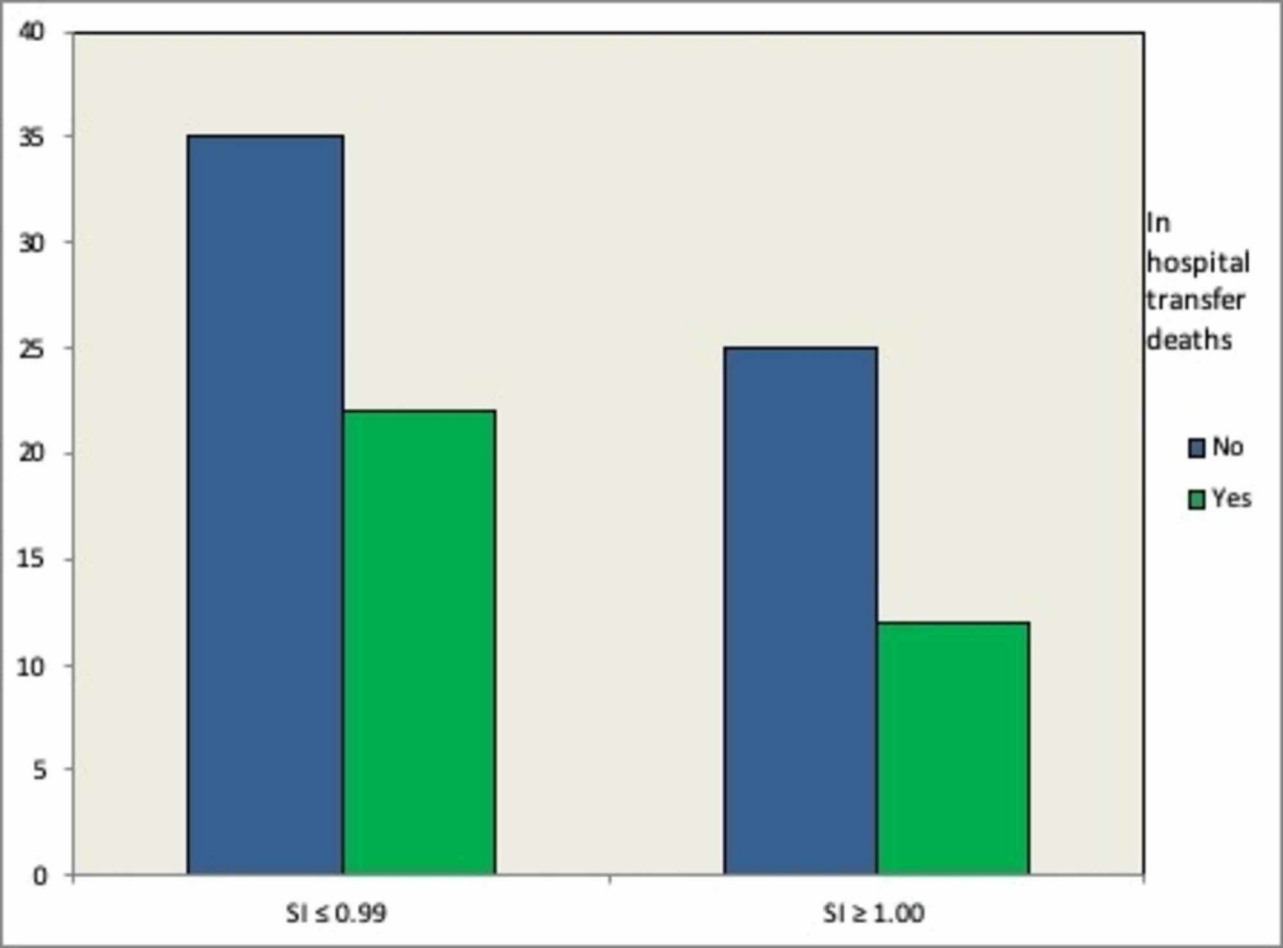
In-hospital transfers to the medical intensive care unit SI: shock index

A total of 1,001 patient records were reviewed. However, only 950 patients were included in the study as the rest had incomplete records (Table 1). Seven hundred and forty-three patients were found to have a SI ≤ 0.99 with a mortality rate of 15.9%. Two hundred and seven patients were found to have an SI ≥ 1.00 with a mortality rate of 22.7%. There was a statistically significant positive correlation between a SI ≥ 1.00 and a higher mortality rate, as analyzed by a Chi-square test (P < .001) (Figure 1). In a subset analysis of the 185 patients on beta-blockers, there was a 17% (25/143) mortality rate in those with a SI ≤ 0.99 and a 26% (11/42) mortality rate in those with a SI ≥ 1.00 (Figure 2). These values, however, did not achieve statistical significance with a p-value of 0.21 (a power analysis was performed showing an adequate number of patients in the analysis). A separate analysis of mortality rates was undertaken for rapid responses on medical floor patients transferred to the ICU (Figure 3). Of the total 84 patients identified who were transferred from the medical floor to the ICU, 57 patients were found to have a SI ≤ 0.99, and of those, there was a mortality rate of 38.6% (22 patients out of 57). Twenty-seven patients were found to have a SI ≥ 1.00, and of those, there was a mortality rate of 44.4% (12 out of 27). This correlation was not statistically significant with a p-value of 0.449.

**Table 1:** Baseline Demographic Characteristics of Patients SI: shock index

Characteristics	Patients (N = 950)	%
Mean age in yrs	63.3	
Patients on beta-blockers	185	19.5
Patients on albuterol	10	1.1
Patients with SI ≤ 0.99	743	78.2
# who expired with SI ≤ 0.99	118	12.4
Patients with SI ≥ 1.00	207	21.8
# who expired with SI ≥ 1.00	47	4.9
Patients transferred from the floor	84	8.8

## Discussion

With many studies examining the benefits of SI in trauma patients and serving as a guide to the degree of hypovolemia following hemorrhage, we were able to extend our data to all patients admitted to the MICU with diagnoses including, but not limited to, diabetic ketoacidosis, status asthmaticus, and respiratory failure. We were able to find that there was a correlation between a higher shock index and mortality rate [7, 10-11]. The SI may be used as a tool in the initial triage, in addition to the patient’s clinical context, to gauge higher acuity earlier without waiting for laboratory values. The analysis shows that a SI value of greater than one is indicative of a higher mortality rate in these patients. The medical floor transfers may not have shown significance due to a very small population. With only 84 transfers documented going to the ICU during the conducted time period, there may have been insufficient data to accurately predict a mortality difference.

Although the study was able to effectively use the SI in MICU patients to predict mortality, there were a few limitations that may have skewed the vital signs used in the calculation of the SI. Many of the patients with asthma and chronic obstructive pulmonary disease (COPD) received albuterol treatment by EMS prior to coming to the emergency department. Albuterol treatment can induce side effects, such as tachycardia secondary to peripheral vasodilation and cardiac stimulation. Therefore, it may have overestimated the true SI. A small number of patients in this study were also administered epinephrine by emergency medical services (EMS) during cardiopulmonary resuscitation prior to presenting to the emergency department. Epinephrine obviously causes both tachycardia and hypertension; therefore, it is hard to estimate an accurate SI in such patients. In addition to this, a very small number of patients may have used recreational drugs, such as cocaine, prior to the emergency department visit, which may have induced tachycardia and hypertension. An important limitation is noted by looking at our data; 476 patients were 65 years or older. Since the elderly are not able to regulate temperature control appropriately, many elderly patients in this study may have been hypothermic. Since we did not identify the core body temperature of patients in this study, it is possible that a few of the patients may have been hypothermic defined by a core body temperature less than 35°Celsius (C). Studies have shown that mild hypothermia (34°C to 35°C) causes an increase in blood pressure and pulse. Moderate (30°C to 34°C) and severe (below 30°C) hypothermia can cause bradycardia and hypotension [12]. Therefore, the true SI in hypothermic patients may have been over or underestimated. Another possible age-related factor that we did attempt to include in our analysis was the use of beta-blockers, which occurred in 19.4% of our study population. Finally, another limiting factor to take into consideration is that many deaths may have occurred secondary to withdrawal of advanced care for the patient due to poor prognosis, promoting natural death. 

## Conclusions

As shown by this data, the SI can be a useful, quick, and reliable tool to predict mortality rates in adult MICU patients when they first arrive at the emergency room, prior to any other lab values. Despite taking into account one of the most limiting factors (beta-blockers), the data still proves significance in mortality rates.
